# Microbleed clustering in thalamus sign in CADASIL patients with *NOTCH3* R75P mutation

**DOI:** 10.3389/fneur.2023.1241678

**Published:** 2023-08-23

**Authors:** Jun Takei, Yujiro Higuchi, Masahiro Ando, Akiko Yoshimura, Jun-Hui Yuan, Natsumi Fujisaki, Takashi Tokashiki, Naomi Kanzato, Manabu Jonosono, Takeshi Sueyoshi, Naoaki Kanda, Hideki Matsuoka, Ryuichi Okubo, Masahito Suehara, Eiji Matsuura, Hiroshi Takashima

**Affiliations:** ^1^Department of Neurology and Geriatrics, Kagoshima University Graduate School of Medical and Dental Sciences, Kagoshima, Japan; ^2^Department of Neurology, National Hospital Organization Okinawa Hospital, Okinawa, Japan; ^3^Department of Neurology, Okinawa Prefectural Southern Medical Center & Children's Medical Center, Okinawa, Japan; ^4^Department of Neurology, Okinawa Chubu Hospital, Okinawa, Japan; ^5^Department of Radiology, Minei Daiichi Hospital, Okinawa, Japan; ^6^Department of Neurology, Imamura General Hospital, Kagoshima, Japan; ^7^Department of Cerebrovascular Medicine, Stroke Center, National Hospital Organization Kagoshima Medical Center, Kagoshima, Japan; ^8^Department of Neurology, Fujimoto General Hospital, Miyazaki, Japan

**Keywords:** CADASIL, *NOTCH3*, R75P, CMBs, thalamus, MCT sign

## Abstract

**Background and objective:**

Cerebral autosomal dominant arteriopathy with subcortical infarcts and leukoencephalopathy (CADASIL) is an inherited cerebral microvascular disease characterized by the development of vascular dementia and lacunar infarctions. This study aimed to identify the genetic and clinical features of CADASIL in Japan.

**Methods:**

We conducted genetic analysis on a case series of patients clinically diagnosed with CADASIL. Clinical and imaging analyses were performed on 32 patients with pathogenic mutations in the *NOTCH3* gene. To assess the presence of cerebral microbleeds (CMBs), we utilized several established rating scales including the Fazekas scale, Scheltens rating scale, and Microbleed Anatomical Rating Scale, based on brain MRI images.

**Results:**

Among the 32 CADASIL patients, 24 cases were found carrying the R75P mutation in *NOTCH3*, whereas the remaining eight cases had other *NOTCH3* mutations (R75Q, R110C, C134F, C144F, R169C, and R607C). The haplotype analysis of the R75P mutation uncovered the presence of a founder effect. A brain MRI analysis revealed that cases with the R75P mutation had a significantly higher total number of CMBs, particularly in the thalamus when compared to patients with other *NOTCH3* mutations. Among 15 out of 24 cases with the R75P mutation, we observed a notable clustering of CMBs in the thalamus, termed microbleed clustering in thalamus sign (MCT sign).

**Conclusion:**

We propose that the MCT sign observed in *NOTCH3* R75P-related CADASIL patients may serve as a potentially characteristic imaging feature. This finding offers further insights into the interactions between genotypes and phenotypes between *NOTCH3* and CADASIL.

## 1. Introduction

Cerebral autosomal dominant arteriopathy with subcortical infarcts and leukoencephalopathy (CADASIL) is a rare hereditary cerebrovascular disease, characterized by vascular dementia and lacunar infarctions (1). Mutations in the *NOTCH3* gene, which encodes notch receptor 3, have been found responsible for CADASIL (2). The majority of pathogenic mutations in CADASIL are found within the epidermal growth factor-like repeat (EGFr) domain of the Notch3 extracellular region, which is encoded by exons 2-24 (3). On the other hand, mutations beyond exon 24 are known to be associated with other conditions, such as myofibromatosis, infantile 2 (4), and lateral meningocele syndrome (5). The typical presentation of CADASIL includes multiple cerebral infarctions in young patients, migraine headaches, depressive symptoms, subcortical dementia, and white matter lesions, especially in the temporal pole, observed on brain MRI.

However, the R75P mutation in *NOTCH3*, which is frequently reported in Korea and Japan, does not exhibit the characteristic temporal pole lesions typically seen in CADASIL (6–8). Instead, it presents images resembling sporadic lacunar infarction, which indicates a unique phenotype and could potentially result in its oversight during routine clinical practice (9). On the other hand, CADASIL is known to be associated with cerebral microbleeds (CMBs) as well (10, 11). With respect to the R75P mutation, there is limited knowledge regarding the frequency, location, and other characteristics of CMBs in the brain MRI of CADASIL patients.

In this study, we conducted a comprehensive analysis of clinical and imaging features from CADASIL patients in the Japanese population, focusing specifically on individuals harboring the R75P mutation in *NOTCH3*, and we provided a further understanding of genotype–phenotype association of CADASIL.

## 2. Materials and methods

### 2.1. Patient selection

We conducted a retrospective analysis of 53 patients from 47 pedigrees who were clinically diagnosed with CADASIL and had a pathogenic *NOTCH3* mutation. The data included patients examined between 2006 and 2021. The initial clinical evaluation of patients was carried out by experienced neurologists at their respective local facilities and was reassessed by a second neurologist in our department. The inclusion was based on their clinical characteristics, information regarding vascular risk factors, and brain MRI images. In this study, we included a total of 32 patients (from 31 pedigrees) who had both clinical and brain MRI data available. Patients who did not have available T2^*^-weighted imaging in their brain MRI were excluded from the study. These patients were enrolled for further analyses.

### 2.2. Genetic analysis

The genomic DNA of patients was extracted from the peripheral blood mainly using a Puregene Blood Kit (QIAGEN, USA) according to the manufacturer's instructions. Exons 3 and 4 of the *NOTCH3* gene were amplified using PCR and analyzed using Sanger sequencing. The R607C mutation was initially identified using whole exome sequencing by a previously described workflow (12) and validated by Sanger sequencing. The Human Gene Mutation Database (HGMD) (https://my.qiagendigitalinsights.com/bbp/view/hgmd/pro/start.php) was used to determine the pathogenicity of all detected variants in *NOTCH3*.

### 2.3. Brain MRI analysis

White matter lesions observed on brain MRI images were evaluated using two different scales, including the Fazekas scale for periventricular and deep white matter regions (13) and the Scheltens rating scale in the external capsule and temporal pole areas (14). The Microbleed Anatomical Rating Scale (MARS) was utilized to analyze the frequency, number, and location of CMBs on T2^*^-weighted images (15).

### 2.4. Haplotype analysis

To access the presence of a founder effect for the hotspot mutation, R75P in the *NOTCH3* gene, we conducted haplotype analysis. A total of twelve markers were employed, consisting of five microsatellite markers (D19S840, D19S415, D19S923, D19S432, and D19S885) and seven single nucleotide polymorphisms (SNPs) (rs4926222, rs2335219, rs4809026, rs7245563, rs141521732, rs6512033, and rs2079234). PCR products of these markers were analyzed using the ABI 3130xL or 3500xL gene analyzer (Applied Biosystems) and Peak Scanner software v1.0 (Thermo Fisher Scientific, Waltham, MA, USA). All primer sequences and information for microsatellite markers were obtained from the National Center for Biotechnology Information (NCBI) database.

### 2.5. Statistical analysis

To evaluate phenotypes associated with the R75P mutation, we categorized the patients into two distinct groups: the R75P mutation group and the group comprising individuals with other *NOTCH3* mutations. The Mann–Whitney U-test was utilized to compare the profiles of CMB between different groups. Statistical analysis was performed with Prism 9 (GraphPad Software), and a two-sided *p*-value of < 0.05 was considered to be statistically significant.

## 3. Results

### 3.1. Genetic analysis

Among the 32 CADASIL patients enrolled in this study, a total of seven pathogenic mutations in the *NOTCH3* gene were detected, including R75P (24 cases), C144F (2 cases; 2 cases from same pedigree), R169C (2 cases), R110C (1 case), C134F (1 case), R607C (1 case), and R75Q (1 case) (Supplementary Table 1).

The 32 patients were subsequently categorized into two groups for analyses: the R75P group (*n* = 24) and the other mutation group (*n* = 8).

### 3.2. Clinical summary

All mutations identified in this study were located within exons 2 to 24, and all cases exhibited the phenotype of CADASIL. There were no phenotypes of lateral meningocele syndrome or myofibromatosis observed in any of our cases. Clinical information of the two groups, the R75P group and the other mutation group, are summarized in Table 1. In the R75P group, it was noted that both age at onset (55.1 ± 8.4 years vs. 50.9 ± 8.4 years) and diagnosis (61.0 ± 7.7 years vs. 54.5 ± 10.4 years) were late, and the frequency of vascular risk factor complications (hypertension, dyslipidemia, and diabetes) was higher than the other mutation group. Cerebral infarction (19/24 and 5/8) and dementia (11/24 and 4/8) were the predominant clinical symptoms observed in both the R75P group and the other mutation group, with no significant difference between the two groups. Migraine was recorded within nine cases, consisting of six cases with R75P mutation and three cases with other mutations. There was a likelihood of cerebral hemorrhage being more common in the R75P group compared to patients with other mutations (5/24 vs. 0/8).

**Table 1:** Comparison of clinical and MRI imaging features between the R75P mutation group and the other mutation group.

**Characteristics**	**All (*n =* 32)**	**R75P (*n =* 24)**	**Other (*n =* 8)**
Age at onset (year, mean ± SD)	54.0 ± 8.4	55.1 ± 8.4	50.9 ± 8.4
Age at diagnosis (year, mean ± SD)	59.4 ± 8.8	61.0 ± 7.7	54.5 ± 10.4
Sex (M/F)	15/17	11/13	2/6
Family history, *n* (%)	28/30 (93.3%)	21/22 (95.5%)	7/8 (87.5%)
**Vascular risk factors**, ***n*** **(%)**			
Hypertension	10/31 (32.3%)	9/23 (39.1%)	1/8 (12.5%)
Dyslipidemia	10/31 (32.3%)	9/23 (39.1%)	1/8 (12.5%)
Diabetes	5/31 (16.1%)	4/23 (17.4%)	1/8 (12.5%)
**Clinical symptoms**, ***n*** **(%)**			
Migraine	9/31 (29.0%)	6/23 (26.1%)	3/8 (37.5%)
Cerebral infarction/TIA	24/32 (75.0%)	19/24 (79.2%)	5/8 (62.5%)
Cerebral hemorrhage^*^	5/32 (15.6%)	5/24 (20.8%)	0/8 (0.0%)
Dementia	15/32 (46.9%)	11/24 (45.8%)	4/8 (50.0%)
Pyramidal signs	17/26 (65.4%)	12/19 (63.2%)	5/7 (71.4%)
Bulbar palsy	12/31 (38.7%)	9/23 (39.1%)	3/8 (37.5%)
Depression	2/32 (6.3%)	2/24 (8.3%)	0/8 (0.0%)
Parkinsonism	8/32 (25.0%)	7/24 (29.2%)	1/8 (12.5%)
Ataxia	4/32 (12.5%)	3/24 (12.5%)	1/8 (12.5%)
**MRI findings**			
PVH: median of Fazekas scale (IQR)	3 (2.75–3)	3 (3)	3 (2–3)
WMH: median of Fazekas scale (IQR)	3 (3)	3 (3)	3 (3)
PVH (prevalence), *n* (%)	32/32 (100%)	24/24 (100%)	8/8 (100%)
WMH (prevalence), *n* (%)	32/32 (100%)	24/24 (100%)	8/8 (100%)
**Temporal pole lesion: median of scheltens scale (IQR)**	0 (0–5)	**0 (0–0)**	6 (5.75–6)
External capsule lesion: median of Scheltens scale (IQR)	5 (5–6)	5 (4.5–6)	5 (5–5.25)
**Temporal pole lesion (prevalence)**, ***n*** **(%)**	12/32 (37.5%)	**4/24 (16.7%)**	8/8 (100%)
External capsule lesion (prevalence), *n* (%)	29/32 (90.6%)	22/24 (91.7%)	7/8 (87.5%)

TIA, transient ischemic attack; PVH, periventricular hyperintensity; WMH, white matter hyperintensity; IQR, interquartile range;

* Symptomatic only. Bold highlights temporal pole lesions in the R75P group.

### 3.3. Brain MRI analysis

The brain MRI findings from 32 patients are shown in Table 1 and Supplementary Table 1, including the scales evaluated using Fazekas, Scheltens, and MARS. Moreover, Table 2 displays the number of CMBs by an anatomic site along with their respective percentages of the total CMB number. In addition to comparing the R75P group with the other mutation group, we conducted a separate analysis by comparing patients who carried the R75P mutation but did not have hypertension.

**Table 2:** Anatomical distribution of CMBs among the R75P and R75P without hypertension and other mutation groups.

**Distribution, *n* (%)**	**Total (*n =* 32)**	**R75P (All, *n =* 24)**	**R75P without hypertension (*n =* 14)**	**Other (*n =* 8)**
Brainstem	65/1,055 (6.2%)	58/942 (6.2%)	23/506 (4.5%)	7/113 (6.2%)
Cerebellum	67/1,055 (6.4%)	64/942 (6.8%)	13/506 (2.6%)	3/113 (2.7%)
Basal ganglia	94/1,055 (8.9%)	90/942 (9.6%)	50/506 (9.9%)	4/113 (3.5%)
**Thalamus**	218/1,055 (20.7%)	**206/942 (21.9%)**	**117/506 (23.1%)**	12/113 (10.6%)
Internal capsule	17/1,055 (1.6%)	17/942 (1.8%)	3/506 (0.6%)	0/113 (0.0%)
External capsule	7/1,055 (0.7%)	6/942 (0.6%)	3/506 (0.6%)	1/113 (0.9%)
Corpus callosum	5/1,055 (0.5%)	5/942 (0.5%)	4/506 (0.8%)	0/113 (0.0%)
DPWM	29/1,055 (2.7%)	27/942 (2.9%)	13/506 (2.6%)	2/113 (1.8%)
**Frontal**	78/1,055 (7.4%)	61/942 (6.5%)	37/506 (7.3%)	**17/113 (15.0%)**
Parietal	122/1,055 (11.6%)	106/942 (11.3%)	71/506 (14.0%)	16/113 (14.2%)
**Temporal**	265/1,055 (25.1%)	**222/942 (23.6%)**	**128/506 (25.3%)**	**43/113 (38.1%)**
Occipital	84/1,055 (8.0%)	77/942 (8.2%)	41/506 (8.1%)	7/113 (6.2%)
Insula	4/1,055 (0.4%)	3/942 (0.3%)	3/506 (0.6%)	1/113 (0.9%)
Infratentorial	132/1,055 (12.5%)	122/942 (13.0%)	36/506 (7.1%)	10/113 (8.8%)
Deep	370/1,055 (35.1%)	351/942 (37.3%)	190/506 (37.5%)	19/113 (16.8%)
Lobar	553/1,055 (52.4%)	469/942 (44.2%)	280/506 (55.3%)	84/113 (74.3%)

DPWM, deep periventricular white matter. Bold indicates the top 2 locations with multiple CMBs.

**Figure 1:**
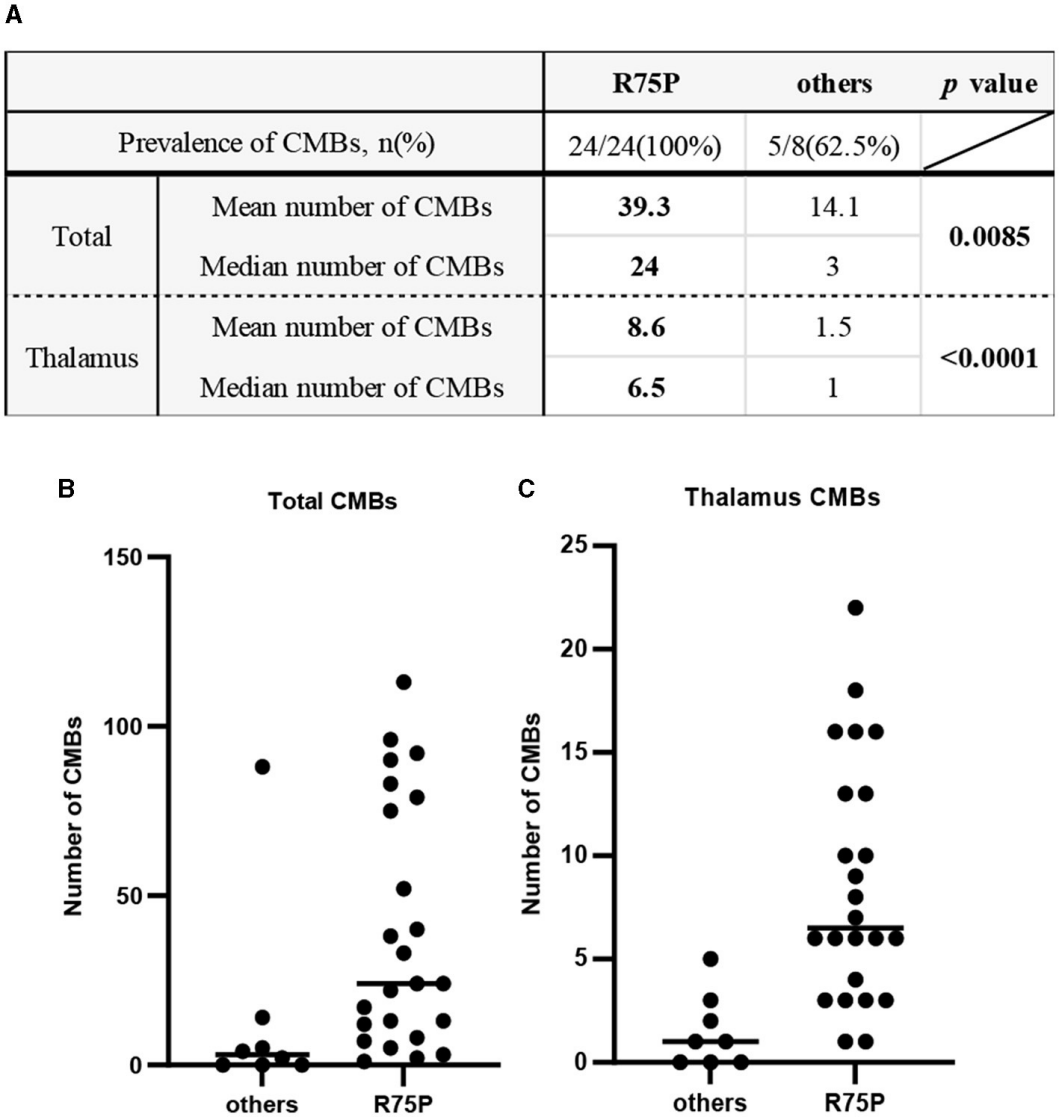
Prevalence and number of CMBs between the R75P group and the other mutation group. **(A)** Mean and median numbers of CMB in the total brain and the thalamus region are listed. Dot plots show the CMB numbers of each patient between two groups in the total brain **(B)** and the thalamus region **(C)**. Bars indicate median values.

The prevalence of CMBs was found in 100% of cases with the R75P mutation (24/24), whereas it was only observed in 62.5% of cases with other mutations (5/8) (Figure 1A). Mean CMB numbers in the R75P and other mutation groups were 39.3 (median = 24) and 14.1 (median = 3.0), respectively, which suggested a significant difference (*p* = 0.0085). Moreover, given the higher occurrence of CMBs in the thalamus region within the R75P group, we conducted an additional analysis focusing on the mean and median number of CMBs per patient in the thalamus. The analysis revealed a mean of 8.6 CMBs (median = 6.5) in the R75P group, which was found to be statistically higher than that of the other mutation group, with a mean of 1.5 (median = 1.0) (Figures 1A–C).

### 3.4. Haplotype analysis

Haplotype analysis was conducted on 16 unrelated patients carrying R75P mutation in the *NOTCH3* gene (Table 3). The analysis using 12 microsatellites and SNP variants uncovered a shared haplotype block of approximately 22.5 kbp spanning the locus between D19S923 (GRCh37; chr19:15297088~15297432) and rs141521732 (GRCh37; chr19:15319920).

**Table 3:** Haplotype analysis of 16 unrelated CADASIL patients with R75P mutation in *NOTCH3*.

**rsID/Marker**	**Locus (Mb)**	**P4**	**P18**	**P25**	**P32**	**P29**	**P27**	**P12**	**P16**	**P5**	**P10**	**P28**	**P22**	**P13**	**P14**	**P23**	**P31**
D19S840	13.8	197/197	197/209	197/207	197/207	197/195	197/197	197/209	197/209	197/207	207/207	197/207	197/197	197/209	207/207	197/207	205/207
rs4926222	14.6	G/A	G/A	G/A	G/A	G/A	G/A	G/G	G/A	G/A	G/A	G/A	G/G	G/A	G/G	G/G	G/A
rs2335219	14.9	G/G	G/G	G/G	G/T	G/G	G/T	G/G	G/G	G/G	G/T	G/G	G/G	G/T	G/G	G/G	G/G
D19S415	15.0	252/252	250/250	250/250	250/250	250/250	250/250	250/250	250/250	250/250	250/250	250/250	250/250	250/250	250/250	250/250	250/250
rs4809026	15.3	C/C	C/C	C/C	C/C	C/C	C/C	G/G	G/G	G/G	G/G	G/G	G/G	G/G	G/G	G/G	G/G
**D19S923**	**15.3**	152/**154**	152/**154**	152/**154**	152/**154**	152/**154**	152/**154**	152/**154**	162/**154**	**154/154**	152/**154**	152/**154**	152/**154**	152/**154**	152/**154**	**154/154**	**154/154**
rs7245563	15.3	**C**/T	**C**/T	**C**/T	**C**/T	**C**/T	**C**/T	**C/C**	**C/C**	**C/C**	**C/C**	**C/C**	**C/C**	**C/C**	**C/C**	**C/C**	**C/C**
R75P	15.3	mt/wt	mt/wt	mt/wt	mt/wt	mt/wt	mt/wt	mt/wt	mt/wt	mt/wt	mt/wt	mt/wt	mt/wt	mt/wt	mt/wt	mt/wt	mt/wt
**rs141521732**	**15.3**	**C/C**	**C/C**	**C/C**	**C/C**	**C/C**	**C/C**	**C**/T	**C/C**	**C/C**	**C**/T	**C/C**	**C/C**	**C/C**	**C/C**	**C/C**	**C/C**
rs6512033	15.6	A/A	C/C	C/C	C/A	C/C	C/A	C/A	C/A	C/C	C/A	C/A	C/A	C/A	C/C	A/A	A/A
D19S432	15.7	181/181	197/197	181/181	181/181	181/181	181/181	181/201	181/181	181/197	181/181	181/181	181/181	185/197	185/197	181/197	181/197
rs2079234	15.9	T/T	T/T	T/G	T/G	T/G	T/T	T/T	T/G	T/T	T/G	T/G	G/G	T/T	T/T	T/T	T/G
D19S885	16.2	173/184	169/169	184/184	169/173	169/184	169/173	169/173	169/173	184/173	169/173	169/169	184/184	173/173	169/173	173/173	169/173

P, Patient; mt: mutant; wt: wild type. Bold highlights the shared haplotype block spanning the genetic locus between D19S923 and rs141521732.

## 4. Discussion

This study presents a case series of genetically diagnosed Japanese patients with CADASIL, with a specific focus on their clinical manifestations and brain MRI characteristics. The aim is to provide a comprehensive understanding of the disease profile in this specific cohort, shedding light on the clinical presentation and imaging findings associated with CADASIL in the Japanese population. Patients harboring the hotspot mutation R75P in the *NOTCH3* gene exhibit distinct MRI features when compared to individuals with other mutations.

Previous reports have established CADASIL as a significant risk factor for the development of CMBs, along with hypertension, cerebral amyloid angiopathy, and age (10, 11). CMBs are present in 30 to 70% of CADASIL cases and have been frequently detected in the cortical regions, particularly in the thalamus and temporal lobe (10, 16–21). In the present study, it is noteworthy that the prevalence of CMBs in the R75P group was 100%, which is higher than that mentioned in the previous studies. In contrast, the occurrence of CMBs in the other mutation group was observed in only 62.5% of cases, suggesting that individuals with R75P mutation may have a higher propensity for developing CMBs.

**Figure 2:**
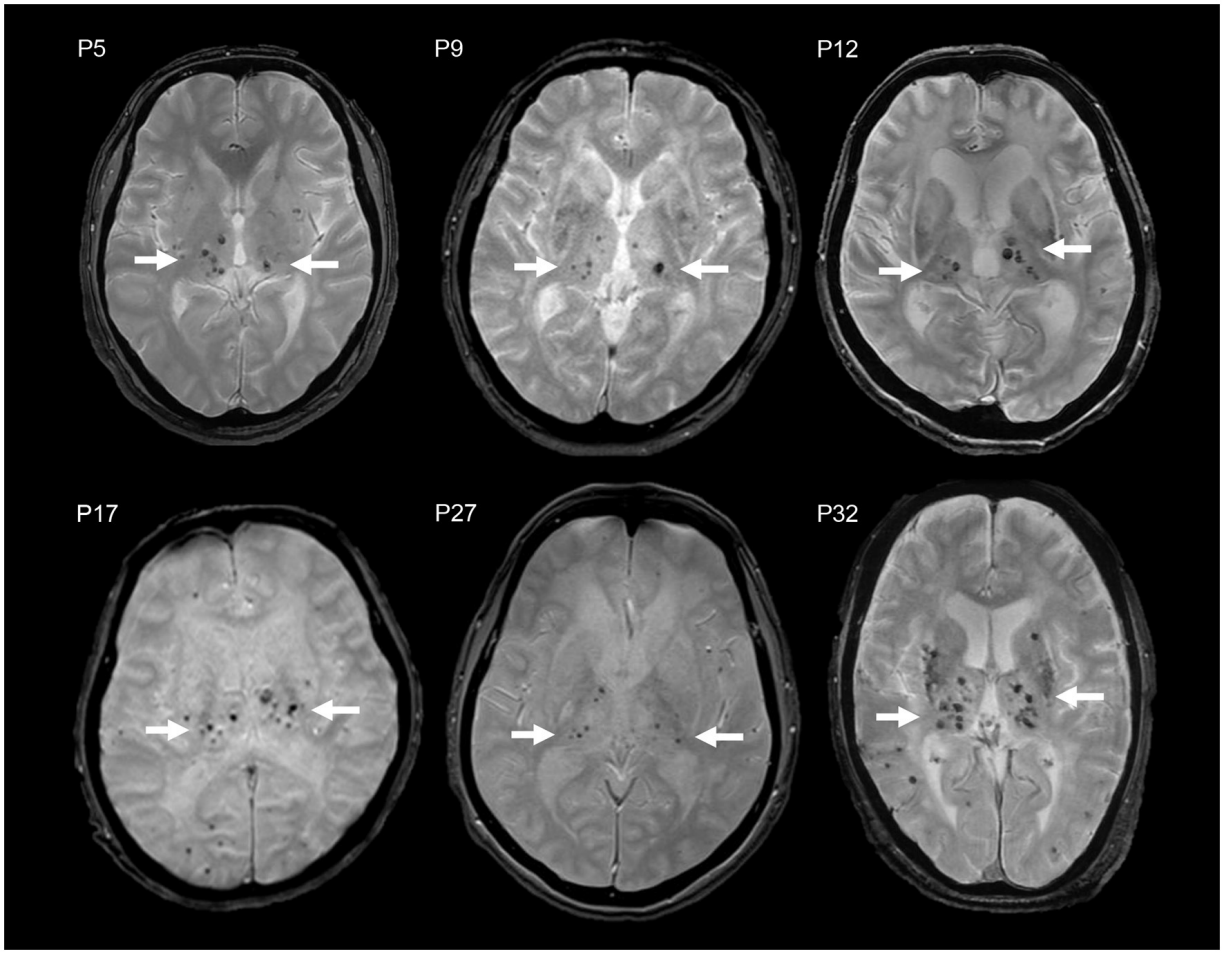
MCT signs in brain MRI T2*WI of six CADASIL patients with R75P mutation (P5, P9, P12, P17, P27, and P32). Multiple CMBs are accumulated in the thalamus (arrow).

Particularly, the R75P group exhibited a higher prevalence and number of CMBs in the thalamus compared to other *NOTCH3* mutations, manifesting a striking imaging pattern of multiple CMBs concentrated within a relatively small anatomical region (Figure 2). We propose to designate the pattern “microbleed clustering in thalamus sign (MCT sign)” and suggest that it may serve as a characteristic feature of the R75P mutation. In the present study, we observed the presence of the MCT sign in 15 out of 24 cases (62.5%) in the R75P group.

Studies on hypertensive stroke patients have reported a variable prevalence of CMBs, ranging from 27 to 71%, which were typically observed in cortical-subcortical regions, the thalamus, and the putamen/pallidum (22, 23). Among these, deep CMBs have been found to be the most prevalent, accounting for approximately 25% of cases, followed by diffuse CMBs at 15% and lobar CMBs at 5% (24). In contrast, cerebral amyloid angiopathy is recognized for its association with CMBs primarily in the cortical areas (25). Interestingly, the distribution of CMBs in CADASIL patients with the R75P mutation showed similarities to those observed in hypertensive stroke patients, making it challenging to differentiate these two conditions based solely on CMB distribution. More importantly, a similar CMB pattern was observed in patients carrying the R75P mutation, even in the absence of hypertension. This finding suggests that the influence of hypertension on the development of CMBs in these patients may be minimal. These findings emphasize the distinct distribution and potential clinical implications of the MCT sign in *NOTCH3* R75P mutation-related CADASIL.

We then proceeded to investigate the significantly higher number of CMBs per patient in the R75P group. Through a reanalysis of the available literature on CMBs in hypertensive stroke patients, the mean number of CMBs per patient was found to range from 1.77 to 2.4 (as shown in Table 4) (22–24). Comparatively, the R75P group in our study exhibited a mean number of CMBs at 39.3 per patient, highlighting the increased burden of CMBs associated with the R75P mutation in CADASIL patients.

**Table 4:** Literature review and comparison of CMB number in patients with hypertensive microangiopathies.

	**Kinoshita et al**. **(****22****) Fan et al**. **(****23****) Yakushiji et al**. **(****24****)**			**Nannucci et al**. **(****26****)**					**This study**	
**Target**	**Hypertensive microangiopathies**			**R133C**	**R141C**	**R169C**	**R182C**	**R607C**	**R75P**	**Other**
**Number**	***n** =* **68**	***n** =* **82**	***n** =* **124**	***n** =* **7**	***n** =* **13**	***n** =* **16**	***n** =* **10**	***n** =* **13**	***n** =* **24**	***n** =* **8**
Prevarence, *n* (%)	48/68 (71%)	22/82 (27%)	NA	3/7 (42.9%)	4/13 (30.8%)	3/16 (18.8%)	0/10 (0.0%)	8/13 (61.5%)	24/24 (100%)	5/8 (62.5%)
Total mean number/per patient (number/patient)	2.0 (137/68)	1.8 (145/82)	2.4 (296/124)	6.9 (48/7)	1.3 (17/13)	0.4 (6/16)	0	3.0 (39/13)	**39.3** (942/24)	14.1 (113/8)
Total median number,/per patient (range)	NA	NA	NA	6.9 (0–28)	1.3 (0–10)	0.4 (0–4)	0	3.0 (0–10)	**24** (1–113)	3 (0–88)
Thalamus mean number, /per patient (number/patient)	NA	NA	NA	3.6 (25/7)	0.5 (6/13)	0.1 (1/16)	0	2.2 (28/13)	**8.6** (206/24)	1.5 (12/8)
Thalamus median number,/per patient (range)	NA	NA	NA	3.6 (0–15)	0.5 (0–4)	0.1 (0–1)	0	2.2 (0–9)	**6.5** (1–22)	1.0 (0–5)

NA, not available. Bold emphasizes the higher number of CMBs in the R75P group.

In addition, we performed a further analysis utilizing supplementary data from the literature, which provided information on CMBs in a cohort of 125 CADASIL cases (Table 4) (26). This analysis focused on comparing the CMB prevalence and the mean and median number of CMBs per patient across different CADASIL variants. None of those variants showed mean and median numbers of CMBs per patient as high as the R75P group in the current study. These findings further underline that the total number of CMBs per patient with the R75P mutation is higher compared to individuals with other mutations in CADASIL.

White matter lesions observed on MRI exhibited a notable prevalence of external capsule lesions in both groups, whereas the R75P group demonstrated a distinctive pattern with fewer temporal pole lesions. This observation aligns with recent reports suggesting that mutations not involving cysteine residues may be associated with a lower frequency of temporal pole lesions (27, 28).

Previous reports on Japanese CADASIL patients have indicated a frequency of 14.8–16.1% for hypertension, 26.2–35.2% for dyslipidemia, and 1.1–4.9% for diabetes as vascular risk factors (29, 30). Although the frequency of vascular risk factor complications in our R75P group was found to be higher compared to the other mutation group, recent reports have indicated that *NOTCH3* mutations are not significantly associated with vascular risk factors (31). Additionally, a comparison between mutations with cysteine residues and variants without cysteine residues has shown no difference in the frequency of vascular risk factors (28).

In addition to cerebral infarction and dementia, migraine was documented in 9 out of 31 cases, which is consistent with previous reports of Japanese CADASIL patients, ranging from 26.9 to 44.3% (8, 29, 32). Cerebral hemorrhage is likely to be more common in patients with the R75P mutation than the patients with other mutations, which supports a single case report suggesting a predisposing of the R75P mutation to cerebral hemorrhage (33). Contrarily, in a separate study on cerebral hemorrhage in CADASIL patients, the number of CMBs was found to be significantly higher in the group with cerebral hemorrhage (34, 35). This finding suggests that the higher frequency of cerebral hemorrhage observed in patients with the R75P mutation may be associated with a greater number of CMBs. In the present study, compared to the cases with R75P, we observed that the only case carrying the R75Q mutation exhibited a similar onset age but presented a relatively milder phenotype (modified Rankin Scale 1). Furthermore, brain MRI, in this case, revealed temporal pole lesions without the presence of CMBs. Literature on the differences in terms of clinical severity and CMBs between R75Q and R75P mutations is limited. Hence, a larger sample size is necessary to demonstrate the genotype–phenotype interactions. On the other hand, it has been noted that the phenotype of CADASIL is highly variable, even within the same family (36). In this study, we also observed diversity in the phenotype and MRI findings between siblings with the C144F mutation (Supplementary Table 1).

Founder effects of multiple *NOTCH3* mutations have been reported in geographically isolated regions, including islands and inland areas such as Taiwan (R544C), Finland (R133C), and central Italy (R607C) (33, 37–39). In this study, the haplotype analysis of 16 unrelated patients spanning the R75P mutation showed a shared haplotype block (approximately 22.5 kbp), suggesting a founder effect of this hotspot mutation in Japan. All 16 patients reside in Kagoshima or Okinawa prefecture, which are both located in the Kyushu Okinawa area of Japan. This geographic clustering of patients from the same region suggests a potential common ancestor or shared genetic background among these individuals.

The analysis conducted in this study utilized MRI images obtained from multiple facilities, resulting in variations in imaging conditions. It is important to note that the detection rate of CMBs can be influenced by technical aspects, such as the strength of the magnetic field (e.g., 1.5 T vs. 3.0 T) and imaging protocols (40, 41). Unfortunately, this study did not specifically consider the potential impact of these technical factors on the detection of CMBs. In addition, considering that susceptibility weighted imaging (SWI) has been reported as a more sensitive sequence for detecting CMBs compared with T2^*^-weighted image (40), it is important to conduct SWI analysis on a larger sample size in the future to validate our findings. Furthermore, due to the absence of hypertensive stroke patients in the present analysis, a direct comparison between patients with hypertensive stroke and those carrying the R75P variant was not available. Indeed, in order to accurately determine the specificity and sensitivity of the MCT sign as a characteristic phenotype for *NOTCH3* R75P-related CADASIL, it is crucial to conduct a study with a larger sample size and a more comprehensive assessment.

NOTCH3-protein-containing deposits known as granular osmiophilic material (GOM) have been recognized as a pathological hallmark in CADASIL (42, 43). A pathological study, which we were unable to conduct in the present study, would provide valuable knowledge regarding the relationship between GOM deposition and CMBs.

Conclusively, we elucidated a remarkably higher prevalence and number of CMBs from a case series of CADASIL patients with R75P mutation in *NOTCH3* compared to the other mutation groups (in-house or studies). We also proposed a potentially characteristic imaging pattern of *NOTCH3* R75P-related CADASIL, referred to as the MCT sign. These findings emphasize the distinct association between the R75P mutation and the presence of CMBs, underscoring the potential importance of this specific mutation in the pathogenesis of CMBs in CADASIL. A genetic test of the *NOTCH3* gene should be considered when an MCT sign is present or the total number of CMBs is high.

## Data availability statement

The original contributions presented in the study are included in the article/Supplementary material, further inquiries can be directed to the corresponding author.

## Ethics statement

This study was approved by the Institutional Review Board of Kagoshima University. All patients provided informed consent to participate in this study and for genetic analysis.

## Author contributions

JT, YH, MA, and HT contributed to the concept and design of the study. JT, YH, AY, and J-HY contributed to the analysis and interpretation of data. NF, TT, NKanz, MJ, TS, NKand, HM, RO, MS, and EM provided the data for patients and participated in the analysis of clinical data. JT produced the original manuscript and all authors approved the final version. HT takes full responsibility for the overall content as the guarantor. All authors contributed to the article and approved the submitted version.
