# Supplementary material for: Microbleed clustering in thalamus sign in CADASIL patients with NOTCH3 R75P mutation
Source: Front Neurol. 2023 Aug 23;14:1241678. doi: 10.3389/fneur.2023.1241678 (PMC10480842; doi:10.3389/fneur.2023.1241678)
Supplement: Supplementary file 1 [file Table_1.DOCX]

**Supplementary Material**

**1 Supplementary Figures and Tables**

**1.1 Supplementary Table**

**Supplementary Table 1.** Detailed clinical and brain MRI records all 32 patients.

HTN: hypertension; DL: dyslipidemia; DM: diabetes mellitus; ICH: intracranial hemorrhage; PVH: periventricular hyperintensity; WMH: white matter hyperintensity; DPWM: deep periventricular white matter; MCT: microbleeds clustering in thalamus; NA: not available.
